# Modification of Different Zirconium Propoxide Precursors by Diethanolamine. Is There a Shelf Stability Issue for Sol-Gel Applications?

**DOI:** 10.3390/ijms10114977

**Published:** 2009-11-13

**Authors:** Gerald I. Spijksma, Dave H. A. Blank, Henny J. M. Bouwmeester, Vadim G. Kessler

**Affiliations:** 1 Inorganic Materials Science, Faculty of Science & Technology, and Mesa ^+^ Research Institute for Nanotechnology, University of Twente, P.O. Box 217, 7500 AE Enschede, The Netherlands; E-Mails: Gerald.Spijksma@stork.com (G.I.S.); D.H.A.Blank@utwente.nl (D.H.A.B.); H.J.M.Bouwmeester@tnw.utwente.nl (H.J.M.B.); 2 Department of Chemistry, SLU, Box 7015, 75007 Uppsala, Sweden

**Keywords:** precursor chemistry, molecular structure, nona-coordinated zirconium

## Abstract

Modification of different zirconium propoxide precursors with H_2_dea was investigated by characterization of the isolated modified species. Upon modification of zirconium *n*-propoxide and [Zr(O^n^Pr)(O^i^Pr)_3_(^i^PrOH)]_2_ with ½ a mol equivalent of H_2_dea the complexes [Zr_2_(O^n^Pr)_6_(OCH_2_CH_2_)_2_NH]_2_ (**1**) and [Zr_2_(O^n^Pr)_2_(O^i^Pr)_4_(OCH_2_CH_2_)_2_NH]_2_ (**2**) were obtained. However, ^1^H-NMR studies of these tetranuclear compounds showed that these are not time-stable either in solution or solid form. The effect of this time instability on material properties is demonstrated by light scattering and TEM experiments. Modification of zirconium isopropoxide with either ½ or 1 equivalent mol of H_2_dea results in formation of the trinuclear complex, Zr{η^3^μ_2_-NH(C_2_H_4_O)_2_}_3_[Zr(O^i^Pr)_3_]_2_(iPrOH)_2_ (**3**) countering a unique nona-coordinated central zirconium atom. This complex **3** is one of the first modified zirconium propoxide precursors shown to be stable in solution for long periods of time. The particle size and morphology of the products of sol-gel synthesis are strongly dependent on the time factor and eventual heat treatment of the precursor solution. Reproducible sol-gel synthesis requires the use of solution stable precursors.

## 1. Introduction

The alkoxides of zirconium are widely used as precursors in the preparation of oxide materials for various applications, ranging from porous membranes [1–3] and matrices for catalysts [4,5] to dense dielectric and ferroelectric thin films for electronic devices [6,7]. In spite of their common use, neither the homometallic nor the heterometallic zirconium alkoxide complexes have been explored sufficiently.

Since their application in, for instance, MOCVD (Metal Organic Chemical Vapor Deposition) or sol-gels may be complicated due to extreme sensitivity to hydrolysis and pyrolysis, zirconium precursors are often modified. A general method to moderate reactivity is the exchange of the alkoxide ligands by chelating organic ligands [8]. In sol-gel studies, acetic acid and acetylacetone are most often applied for this purpose [9]. Acetic acid has the undesired ability to react with alcohol upon release of water and in recent work we showed [10] that the stabilizing effect with acetylacetone is time dependent and disappears completely beyond addition of 1 mol equivalent. It was also shown that the destabilization occurs rapidly and that there can be a tremendous effect on the prepared materials [10]. Complex solution transformations have been revealed even on modification of zirconium and hafnium alkoxides with branched β-diketones, for example, 2,2,6,6-tetramethylheptanedione (Hthd) [11]. The structures of intermediate species formed in solution via modification of zirconium and hafnium alkoxides by acetylacetone and carboxylic acids have recently also been investigated by Bertagnolli *et al*. by EXAFS techniques, revealing possible increase of coordination numbers for these species, compared to initial complexes [12–14]. The stability of formed modified precursor should be considered a relevant issue. For MOCVD shelf stability is an obvious requirement, but even for sol-gel applications where the modification is performed *in situ* its influence might not be negligible.

In our search for a stable modified zirconium precursor we have used diethanolamine, H_2_dea, as a stabilizing agent. To the best of our knowledge, no reports have been published on zirconium precursor structures formed upon modification with H_2_dea; however, the structure of zirconium *n*-propoxide modified with 0.5 mol equivalent of *N*-methyldiethanolamine has been reported [15].

In the present study we attempted to prepare an analogous compound through the modification of zirconium *n*-propoxide with the more common H_2_dea. In addition, other zirconium propoxide precursors, including zirconium isopropoxide and a mixed ligand precursor [Zr(O^n^Pr)(O^i^Pr)_3_(^i^PrOH)]_2_ were modified in an attempt to reach better understanding of the chemistry involved upon modification with H_2_dea. In addition to isolation and structural characterization of the modified precursors, their stability with time and the possible influence on material properties was evaluated.

## 2. Results and Discussion

**Figure 1.**
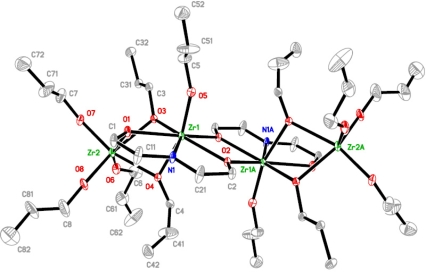
Molecular structure of [Zr_2_(O^n^Pr)_6_(OCH_2_CH_2_)_2_NH]_2_ (**1**).

**Figure 2.**
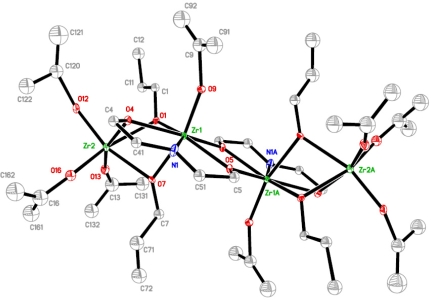
Molecular structure of [Zr_2_(O^n^Pr)_2_(O^i^Pr)_4_(OCH_2_CH_2_)_2_NH]_2_ (**2**).

The structures of the complexes **1** and **2** (Figures 1 and 2 respectively), formed upon modification of zirconium *n*-propoxide and the mixed ligand derivative, resemble closely the structure that Gainsford *et al*. [15] obtained upon modification of zirconium *n*-propoxide with *N*-methyldiethanolamine. Both complexes **1** and **2** are tetranuclear and centrosymmetric, and thus possess two different types of positions for the zirconium atoms. The terminal zirconium atoms are hexa-coordinated through three terminal and three doubly bridging alkoxide ligands, two from *n*-propoxide groups and one from dea. The other position is filled with a heptacoordinated zirconium that is surrounded with one terminal and five doubly bridging alkoxide ligands, two from *n*-propoxide groups and three from dea, and a nitrogen atom from the H_2_dea ligand. The different surrounding of the nitrogen atom is likely to cause some difference in bonding lengths, though they are marginal (Zr-N 2.363(7), 2.35(2), 2.411 Å for respectively **1**, **2** and the complex in [15]). There is no noticeable influence of nitrogen coordination on distances to the bridging alkoxide ligands connected to the modifying agent ((Zr-O 2.211(6), 2.153(14), 2.202 Å for **1**, **2**, respectively, and the complex in [15]).

The most significant difference between **1** and **2** lies in the presence of the more bulky isopropoxide ligands in **2**. They are placed exclusively in the terminal positions. The *n*-propoxide ligands remain bridging. The presence of smaller (*n*-chain structure) bridging ligands appears to be a critical condition for the isolation of this type of molecular structure. The yield of compound **2** is significantly lower than that of **1** for analogous synthesis. This can either be due to higher solubility in the hydrocarbon solvent or to lower stability in solution. The latter will be discussed below.

**Figure 3.**
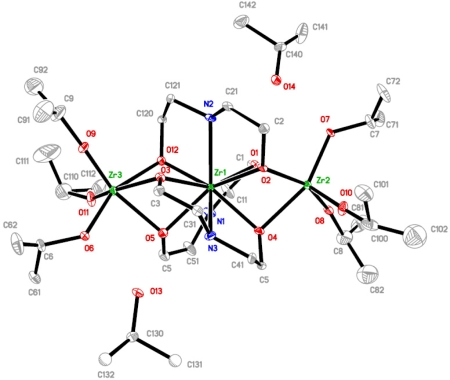
Molecular structure of Zr{η^3^μ_2_-NH(C_2_H_4_O)_2_}_3_[Zr(O^i^Pr)_3_]_2_(^i^PrOH)_2_ (**3**).

The molecular structure of complex **3** (see Figure 3), obtained up on modification of zirconium isopropoxide with either ½ or 1 mol equivalent of H_2_dea, Zr{η^3^μ_2_-NH(C_2_H_4_O)_2_}_3_[Zr(O^i^Pr)_3_]_2_(^i^PrOH)_2_, is completely different from **1** and **2** and rather unique. It is a trinuclear complex with isopropoxide ligands only present at the terminal metal positions. Zirconium atoms in this complex are situated in two different types of position, a nona-coordinated central and two hexa-coordinated terminal ones. The existence of a nona-coordinated zirconium has only been reported under aqueous conditions [16–18], and in heterometallic zirconium-titanium analogs of **3**, reported by us recently [19].

The aqueous media derived compound described by Harben *et al.* [16] has rather uniform bond lengths (*i.e.*, Zr–N 2.225, 2.228 Å; Zr–OH_2_ 2.261 Å; Zr–O–C 2.244, 2.248, 2.242, 2.240 Å; Zr–O–N 2.126, 2.123 Å), indicating a high degree of ionic bonding. Complex **3** on the contrary is significantly more covalently bonded, indicated by predominantly shorter Zr-O bond lengths (*i.e*., 2.193(9), 2.202(9), 2.207(8), 2.206(8), 2.231(9), 2.250(9) Å), while the Zr-N bond lengths are longer (*i.e*., 2.375(10), 2.447(11), 2.485(11) Å) involving much weaker, supposedly predominantly electrostatic, interactions. There is no direct analogy between the complex by Harben *et al*. [16] and **3**, thus **3** should be considered as a representative of a new class of nona-coordinated zirconium compounds.

**Figure 4.**
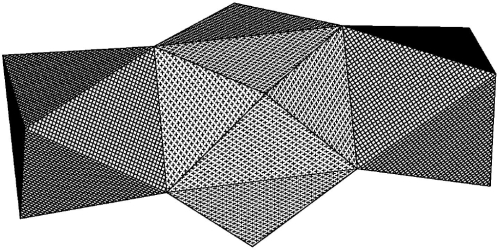
Polyhedral presentation of the molecular structure of **3**.

The structure of **3** gave us an idea for the preparation of new heterometallic precursors. The presence of two types of positions, a nona-coordinated and a hexa-coordinated, provides the possibility to construct new species via self-assembly according to the Molecular Structure Design Concept [20–22]. For example, the hexa-coordinated position appears to be able to host other, smaller, atoms than zirconium. In a preliminary communication [19], we have recently reported the application of this model to the preparation of a new Zr-Ti complex. The molecular structure of the complex obtained was studied by X-ray crystallography and characterized as Zr{η_3_μ_2_-NH(C_2_H_4_O)_2_}_3_[Ti(O^i^Pr)_3_]_2_. This heterometallic complex, derived from **3** [19], has a central Zr atom, with coordination in a very regular tri-capped trigonal prism (see Figure 4) with the oxygen atoms composing the vertices if the prism (Zr(1)-O(2) 2.196(3), Zr(1)-O(3) 2.199(3) and Zr(1)-O(4) 2.210 (3) Å) and the nitrogen atoms as capping vertices (Zr(1)-N(1) 2.440(6), Zr(1)-N(2) 2.439(5) Å). The coordination of the outer Ti atoms is a trigonally distorted octahedra with, in principle, only two types of Ti-O distance: bridging (Ti-O 2.137(3)-2.147(3) Å) and terminal (Ti-O 1.836(5)-1.853(4) Å).

The tri-capped trigonal prismatic coordination of the central zirconium in complex **3** is slightly less regular. This is due to the smaller polarising effect of the outer zirconium atoms compared to titanium, different placement of the molecules in relation to the crystallographic elements of symmetry–triclinic for **3**, the molecule occupying a general position, and tetragonal in the heterometallic complex with the Zr atom placed on a 2(1) axis–and the presence of hydrogen-bonded isopropanol. The hydrogen bonding clearly leads to elongation of one of the Zr-O bonds at each side of the molecule; Zr(1)-O(4) 2.250(9) Å and Zr(1)-O(5) 2.231(9) Å compared to shorter Zr(1)-O(1) 2.207(8), Zr(1)-O(12) 2.193(9), Zr(1)-O(3) 2.202(9) and Zr(1)-O(2) 2.206(8) Å. The influence of the hydrogen-bonded alcohol is also noticeable for the geometry of the outer zirconium atoms. The trigonally distorted octahedron has one longer bridging Zr-O bonding due to each hydrogen-bonded alcohol (2.252(9) Å and 2.287(9) Å against 2.170(9)–2.201(8) Å). The presence of solvating isopropanol molecules is confirmed by the presence of a broad ν-OH band at 3,383 cm^−1^ in the IR spectra.

**Figure 5.**
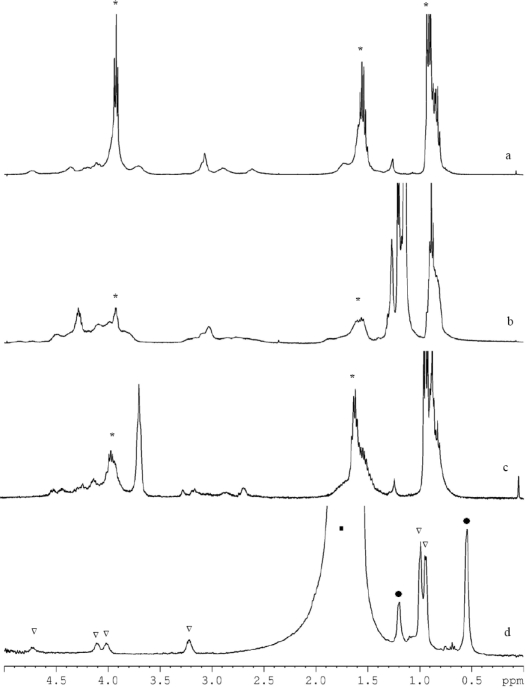
^1^H NMR spectra of the freshly prepared **1** (a) and **2** (b); and of crystals of **1** aged for 3 weeks at r.t. (c), and a fresh sample of **3** (d) in toluene as solvent. The signals assigned to the *n*-propoxide are marked with (*), to toluene and hexane with (▪) and (*), and those assigned to **3** with (^∇^) respectively.

We have carried out an investigation of the time stability in solution as well as in the solid of three isolated compounds. The ^1^H-NMR spectra of the freshly prepared samples of **1** and **2** in deutero-chloroform (Figures 5a and b for **1** and **2**, respectively) can be rationalized as corresponding to the molecular structures observed in the solid state. The spectrum of **1** (Figure 5a) clearly shows the presence of *n*-propoxide ligands (marked with *), the signals at 0.9, 1.52 and 3.93 ppm are respectively assigned to *CH_3_*, *CH_2_* and *OCH_2_*. The intensities are in accordance to what is expected, except for the *CH_3_* signal which overlaps with that of some remaining hexane. The signal of *CH_2_N* of the diethanolamine is at 2.6, 2.88 and 3.94 ppm; the presence of other peaks is in agreement with the XRD data where different N-C bond lengths were also found. The signals of *CH_2_O* of the diethanolamine are poorly resolved between 3.7 and 4.4 ppm. However, the intensity of the signals is comparable with that of the *CH_2_N* signals and close to the expected ratio of 1:3 with the signal of *OCH_2_* at 3.93 ppm.

The spectrum of **2** (Figure 5b) is less well resolved, albeit that the differences and concurrences with **1** can clearly be seen. The *n*-propoxide (marked with *) and isopropoxide ligands are approximately in the ratio 1:2, as in complex **2**. Precise determination of this ratio is inaccurate due to overlap with the hexane signal. The intensity of *CH_2_*, around 1.6 ppm, is the same as that of the unresolved signals of *NCH_2_* between 2.5 and 3.5 ppm. The unresolved peaks between 4.05 and 4.5 have an area three times larger than that of the *NCH_2_*, and may then be assigned to the presence of the *OCH_2_* of the *n*-propoxide and diethanolamine and the *CH* of the isopropoxide.

The spectra recorded after 16 days displayed dramatic changes for both complexes, indicating that both complexes are subject to rearrangements. Initially such rearrangements were thought to be caused by the solvent. Halide-containing solvents such as CDCl_3_ can alkylate amines via the Hoffman reaction. However, the spectra of a fresh sample of crystals aged for three weeks at room temperature showed an analogous spectrum as displayed in Figure 5c. The most striking difference between this spectrum and the initial (Figure 5c) one is the significant increase of the peak at 3.6 ppm and the broadening of the *n*-propoxide peaks (marked with *). The former is, as could be seen from two-dimensional ^1^H-^13^C HMQC spectroscopy, according to the appearance of *OCH_2_* of *n*-propoxide in a bridging position; supporting a possible rearrangement of **1** towards an *n*-propoxide analogue of **3** upon the formation of zirconium *n*-propoxide with a M_4_O_16_ [23] type of structure (as shown in Equation 1)

(1) $$6\;{\left[{\text{Zr}}_{2}{\left({\text{O}}^{\text{n}}\text{Pr}\right)}_{6}{\left({\text{OCH}}_{2}{\text{CH}}_{2}\right)}_{2}\text{NH}\right]}_{2}\;\to \;4\text{Zr}{\left\{\eta^{3}\mu_{2}-\text{NH}{\left({\text{C}}_{2}{\text{H}}_{4}\text{O}\right)}_{2}\right\}}_{3}{\left[\text{Zr}{\left({\text{O}}^{\text{n}}\text{Pr}\right)}_{3}\right]}_{2}\;+\;3\;{\text{Zr}}_{4}{\left({\text{O}}^{\text{n}}\text{Pr}\right)}_{16}$$

It can be concluded that complexes **1** and **2** are not stable in time. For complex **3** a rapid change in structure occurs in deuterochloroform, resulting in precipitation of an insoluble polymer. No precipitations occurred when **3** was dissolved in toluene; the corresponding spectra did not change for several days (Figure 5d). Since the spectra were recorded in non-deuterated toluene, some peaks of the complex may overlap with those of the solvent.

The signals corresponding to toluene and hexane are marked with (▪) and (*), respectively, and those assigned to **3** with (^∇^). The signals at 3.22, 4.02 and 4.12 ppm are assigned to the *CH_2_* of the diethanolamine ligands, the one at 3.22 ppm to *NCH_2_* and the later two to *OCH_2_*. The splitting of the signal assigned to *OCH_2_* may be a result of the longer bond lengths due to hydrogen-bonded isopropanol (see above). However, in the solid state the ratio between longer and normal bond lengths is 2:4, while dissolved in solution it seems to be more like 3:3. The signal at 4.72 ppm that is assigned to the *CH* of the isopropoxide ligands and the solvating isopropanol is in a ratio of 8:12 to that of *NCH_2_*, which is in accordance with the molecular structure. An analogous spectrum was obtained after storing the sample for several days at room temperature, demonstrating the time stability of complex **3**.

However can this time stability of complex **3** really be considered a beneficial property, with respect to the preparation of materials? In case of application in MOCVD and related techniques the absence of shelf stability clearly will influence the formed materials. The influence on sol-gel processed materials is not obvious, since the modification is *in situ.* The influence is evaluated by comparing the properties of gels by light scattering and TEM. The gels were obtained from the sols prepared from zirconium *n*-propoxide modified with 1/2 equivalent mol H_2_dea.

The only difference between the two samples was the refluxing after addition of the modifier, which step is often applied in sol-gel syntheses and accelerates possible transformations. The first indication that the refluxing had influence was observed after the addition of the nitric acid/propanol mixture. The sample that had not been refluxed turned almost instantly into a colorless gel, while the refluxed sample was a clear yellowish sol. The latter sol became a yellowish gel 24 hours later.

**Figure 6.**
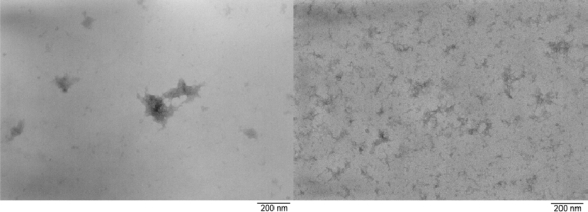
TEM view of jellified sols of zirconium *n*-propoxide modified with ½ mol equivalent of H_2_dea without (left) and with (right) refluxing before hydrolysis.

The light scattering experiments on the gelated sols also showed a difference: the sample that not had been refluxed consisted of particles with an average size of ~300 nm, while the refluxed sample consisted of two types of particles–those in the order of 40 nm (between 10 and 30% of the sample) and larger particles ranging from 130–150 nm. The TEM analysis is consistent with this observation by light scattering. Figure 6 represents the typical appearance of the two samples.

It can clearly be seen that the presence or absence of refluxing has a great influence on the sol and gel properties. Thus even a short time storage, *e.g.*, during synthesis, may cause considerable effects for the synthesis of materials for high-tech applications. Since complex **3** is stable in time, it can be considered as an interesting precursor for the preparation of materials. Another advantage of **3** over **2** and other mixed alkoxide precursors, might be the presence of only one type of alkoxide ligand attached to a unique zirconium atom. Different alkoxides groups or alkoxides attached to differently coordinated metal atoms will differ in reactivity, which can have a significant influence on the structure of the materials formed.

The results obtained in this study have provided also new insight into the possibility of design and application of related heterometallic complexes. The apparent difference in coordination modes for the two sites in the molecule of **3**, one central nona-coordinated position and two peripheral octahedrally coordinated ones, indicated possibility to use this structure type for incorporation of two different types of metal atoms. We have used this approach successfully for the synthesis of shelf stable bimetallic Zr-Ti [19], and Hf-Ti [24] precursors compounds with the general formula M{η^3^μ_2_-NH(C_2_H_4_O)_2_}_3_[M’(OiPr)_3_]_2_, where M = Zr, Hf; M’ = Ti. Their most important feature in comparison with zirconium and hafnium alkoxides modified by simple chelating ligands such as β-diketonate and carboxylate ones [25], is that in the analogues of **3** the chelating heteroligands are “concentrated” at a metal atom within a molecule of heterometallic alkoxide and this permits to keep them evenly distributed on molecular level even in the course of the further sol-gel transformation. Resulting gels are still containing the molecules of organic ligands within their structure and not only on the surface of the oxide nanoparticles, MTSALs [26], resulting from hydrolytic treatment of metal alkoxides [27]. The films, deposited from the sols obtained from such precursors demonstrate, after the appropriate thermal treatment, an important and reproducible fraction of microporosity, resulting most probably from the thermal removal of the residual heteroligands. The obtained coatings have displayed significant difference in dead-end permeance for hydrogen and light hydrocarbons making them attractive candidates for development of microporous membranes for gas separation [28].

## 3. Experimental Section

All manipulations were carried out in a dry nitrogen atmosphere using the Schlenk techniques or a glove box. Hexane and toluene (Merck, p.a.) were dried by distillation after refluxing with LiAlH_4_ and metallic sodium, respectively. Diethanolamine (H_2_dea) was purchased from Aldrich and used without further purification.

IR spectra of nujol mulls were registered with a Perkin Elmer FT-IR spectrometer 1720 X or a Bio-Rad FTS 375 C FT-IR spectrometer equipped with a DTGS detector. ^1^H-NMR spectra were recorded in CDCl_3_ for compounds **1–3** and in toluene for compound **3** solutions on a Bruker 400 MHz spectrometer at 243 K. The results of microanalysis (C, N, H) were obtained by Mikrokemi AB, Uppsala, Sweden, using the combustion technique, and the results of the analysis were in agreement with the expected for the obtained complexes. The particle size distribution was measured by dynamic light scattering (ZetaSizer 3000HSa, Malvern, UK). The transmission electron microscopy (TEM) experiments were performed on a PHILIPS CM30 Twin/STEM.

### 3.1. Synthesis and Sample Preparation

The zirconium propoxide precursors used in this work as starting materials are zirconium isopropoxide, ([Zr(O^i^Pr)_4_(^i^PrOH)]_2_ 99.9%), 70 wt% solution of Zr(O^n^Pr)_4_” (both purchased from Aldrich) and [Zr(O^n^Pr)(O^i^Pr)_3_(^i^PrOH)]_2_ which was prepared according to a recently developed technique [19,23]. All three different precursors were modified with both ½ and 1 mol equivalent of H_2_dea, however only three cases crystals could be isolated. The exact composition of the synthesized samples **1**, **2** and **3** was established with single X-ray crystallography.

*[Zr_2_(O^n^Pr)_6_(OCH_2_CH_2_)_2_NH]_2_* (**1**). Commercial 70% zirconium *n*-propoxide in *n*-propanol [weight: 1.87 g (4.8 mmol)] was dried under vacuum and re-dissolved in hexane (2 mL). After addition of H_2_dea (0.25 g, 0.24 mmol), the colorless sample was placed overnight in the freezer at −30 °C. Subsequently, the sample was dried under vacuum (0.1 mm Hg). The formed solid product was re-dissolved in hexane (1 mL) and placed in the freezer for crystallization. The next day a significant amount of product had been formed, after several days the solution was decanted and the obtained crystals (1.58 g, yield 75%) identified as **1**. IR, cm^−1^: 1,300 w, 1,280 m, 1,252 m, 1,136 w, 1,085 br, 1,021 vw, 997 w, 973 w, 953 vw, 917 m, 886 m, 862 m, 842 m, 826 sh, 782 s, 758 w, 718 m, 627 sh, 599 sh, 551 br, 478 sh.

*[Zr_2_(O^n^Pr)_2_(O^i^Pr)_4_(OCH_2_CH_2_)_2_NH]_2_* (**2**). The prepared [Zr(O^n^Pr)(O^i^Pr)_3_(^i^PrOH)]_2_ (1.07 g, 2.8 mmol) was dissolved in hexane (2 mL) and H_2_dea (0.14 mL, 0.13 mmol) was added dropwise. The sample was stored overnight in the freezer, before removal of the solvents. After re-dissolving the formed solids in hexane (1 mL) the sample was placed back into the freezer; it took several days before the first crystals were formed. After 23 days the solution was decanted and the obtained crystals (0.62 g, yield 0.51%) were identified as **2**. IR, 1,337 m, 1,302 w, 1,272 w, 1,247 w, 1,143 w, 1,096 w, 1,068 br, 1,015 w, 1,000 w, 971 w, 953 w, 920 m, 888 w, 858 vw, 840 w, 820 w, 721 s, 667w, 657 w.

*Zr{η^3^μ_2_-NH(C_2_H_4_O)_2_}_3_[Zr(O^i^Pr)_3_]_2_(iPrOH)_2_* (**3**). The modification of zirconium isopropoxide with ½ equivalent mol of H_2_dea was performed by dissolving the precursor (0.89 g, 2.3 mmol) in a mixture of hydrocarbons (3 mL, hexane/toluene in a volume ratio of 2:1). After addition of the equivalent amount of H_2_dea the colorless sample was placed overnight in the freezer at −30 °C. Subsequently, the solvents and released isopropanol were removed under vacuum (0.1 mm Hg), and the dried compound was re-dissolved in hexane (1 mL) and returned to the freezer for crystallization. After several weeks of crystallization a product was obtained. The white product was analyzed and was found to consist of two crystal types. Single crystal XRD identified these as Zr{η^3^μ_2_-NH(C_2_H_4_O)_2_}_3_[Zr(O^i^Pr)_3_]_2_(iPrOH)_2_ and unreacted zirconium iso-propoxide [29]. An analogous experiment with addition of 1 mol equivalent H_2_dea was performed in order to obtain pure **3**. The yield was 79% (1.09 g zirconium isopropoxide, 0.30 mL H_2_dea, 1.1 g of compound **3**). IR, 3,383 br, 1,358 sh, 1,338 s, 1,323 s, 1,299 s, 1,272 w, 1,253 s, 1,233 s, 1,218 w, 1,163 s, 1,128 s, 1,109 w, 1,077 br, 1,042 w, 992 s, 961 sh, 926 sh, 898 s, 867 sh, 844 s, 774 w, 750 w, 723 s, 669 s, 625 sh, 594 sh, 564 br, 544 sh, 505 br, 486 w, 474 w, 458 sh, 447 w, 423 w.

Preparation of the sols for TEM analysis was carried out using zirconium *n*-propoxide. The solvent of commercial zirconium *n*-propoxide was removed under vacuum, yielding ~2.5 g of residue that was re-dissolved in *n*-propanol (mol ratio 1:10) with addition of ½ mol equivalent of H_2_dea. The sample was optionally refluxed for half an hour. Subsequently, the obtained modified precursor was hydrolyzed by a mixture of 0.1 M nitric acid and *n*-propanol (volume ratio 1:20, ratio zirconium *n*-propoxide and H_2_O 1:2), resulting in a colorless (without refluxing) or slightly yellowish sol (with refluxing). The samples for light scattering and TEM experiments were prepared from the gelled sols by dissolving ~0.05 g in 20 mL *n*-propanol and subsequent peptization with 0.3 mL 0.05 M nitric acid. The samples were ultrasonically treated for 15 minutes. The particle size of the clear solution was determined by light scattering. For TEM analysis a drop of the solution was deposited on a copper supported carbon grid.

### 3.2. Crystallography

Data collection for all single crystals was carried out at 22 °C on a SMART CCD 1k diffractometer with graphite monochromated MoKα. Structures were solved by direct methods. The coordinates of the metal atoms were obtained from the initial solutions and for all other non-hydrogen atoms found in subsequent difference Fourier syntheses. The structural parameters were refined by least squares fitting using both isotropic and anisotropic approximations. The coordinates of the hydrogen atoms were calculated geometrically and were included into the final refinement in isotropic approximation for all the compounds. All calculations were performed using the SHELXTL-NT program package [30] on an IBM PC.

## 4. Conclusions

Complexes **1** and **2** were obtained upon modification of zirconium *n*-propoxide and mixed ligand precursor with ½ mol equivalent of H_2_dea. However, these tetranuclear compounds were not stable in solution and solid form, and the effect of the absence of this stability on materials was found to be important. The trinuclear complex, Zr{η^3^μ_2_-NH(C_2_H_4_O)_2_}_3_[Zr(O^i^Pr)_3_]_2_(^i^PrOH)_2_ (**3**) obtained upon modification of zirconium isopropoxide with either ½ and 1 equivalent mol of H_2_dea, has a unique nona-coordinated central zirconium atom. This attractive nona-coordination of the central zirconium atom in **3** already led to the development of an analogous titanium-zirconium complex and will lead to a whole new series of interesting heterometallic complexes. Another unique feature of **3** is its solution stability, which is lacking in the modification of zirconium isopropoxide with for instance Hacac, and its time stability both dissolved in solution and in the solid state.

## Figures and Tables

**Table 1.** Crystal data and the diffraction experiments details for compounds **1–3.**

**Chemical composition**	**C_44_H_102_N_2_O_16_Zr_4_**	**C_44_H_102_N_2_O_16_Zr_4_**	**C_36_H_85_N_3_O_14_Zr_3_**
Formula weight	1280.16	1280.16	1057.74
Crystal System	Monoclinic	Monoclinic	Triclinic
Space group	P2(1)/c	P2(1)/c	P-1
μ/mm^−1^	0.693	0.674	0.620
*R*1	0.0543	0.0841	0.0777
*w*R2	0.1239	0.2022	0.1883
*a*/Å	10.708(3)	17.384(4)	12.086(6)
*b*/Å	17.749(4)	20.675(4)	15.180(7)
*c*/Å	16.968(5)	18.166(4)	16.576(9)
*α* /°	90	90	73.136(13)
*β* /°	99.960(7)	90.316(5)	85.788(17)
*γ* /°	90	90	68.52(2)
*V*/Å^3^	3176.3(15)	6529(2)	2706(2)
*T*/K	295(2)	295(2)	295(2)
Z	2	4	2
Number of independent reflections	3392 [R(int) = 0.0766]	4205 [R(int) = 0.0982]	5702 [R(int) = 0.0556]
Number of observed reflections	1909 [I > 2sigma(I)]	1915 [I > 2sigma(I)]	2439 [I > 2sigma(I)]
